# Tensor decomposition-based and principal-component-analysis-based unsupervised feature extraction applied to the gene expression and methylation profiles in the brains of social insects with multiple castes

**DOI:** 10.1186/s12859-018-2068-7

**Published:** 2018-05-08

**Authors:** Y.-H. Taguchi

**Affiliations:** Department of Physics, Chuo University, 1-13-27 Kasuga, Bunkyo-ku, Tokyo, 112-8551 Japan

**Keywords:** Tensor decomposition, Principal component analysis, Feature extraction, Gene expression, Methylation, Social insect

## Abstract

**Background:**

Even though coexistence of multiple phenotypes sharing the same genomic background is interesting, it remains incompletely understood. Epigenomic profiles may represent key factors, with unknown contributions to the development of multiple phenotypes, and social-insect castes are a good model for elucidation of the underlying mechanisms. Nonetheless, previous studies have failed to identify genes associated with aberrant gene expression and methylation profiles because of the lack of suitable methodology that can address this problem properly.

**Methods:**

A recently proposed principal component analysis (PCA)-based and tensor decomposition (TD)-based unsupervised feature extraction (FE) can solve this problem because these two approaches can deal with gene expression and methylation profiles even when a small number of samples is available.

**Results:**

PCA-based and TD-based unsupervised FE methods were applied to the analysis of gene expression and methylation profiles in the brains of two social insects, *Polistes canadensis* and *Dinoponera quadriceps*. Genes associated with differential expression and methylation between castes were identified, and analysis of enrichment of Gene Ontology terms confirmed reliability of the obtained sets of genes from the biological standpoint.

**Conclusions:**

Biologically relevant genes, shown to be associated with significant differential gene expression and methylation between castes, were identified here for the first time. The identification of these genes may help understand the mechanisms underlying epigenetic control of development of multiple phenotypes under the same genomic conditions.

**Electronic supplementary material:**

The online version of this article (10.1186/s12859-018-2068-7) contains supplementary material, which is available to authorized users.

## Background

Organisms often exhibit different phenotypes despite a common genomic background. For example, juveniles and adults frequently have different body plans (e.g., tadpoles and frogs, caterpillars and butterflies, and megalopas and clubs). Nonetheless, juvenile and adult organisms have different sizes or must survive in distinct environments, and these conditions require different phenotypes. More striking examples are castes of social insects, such as ants and bees, which can form two distinct forms: queens and workers, both female [1]. They are usually closely related, but queens and workers have different sizes and lifespans. The mechanism that potentially allows social insects to form castes with distinct body plans is the epigenome [2, 3], which is flexible and can lead to the formation of different phenotypes without genomic alterations. Therefore, it is important to determine the correlation between an epigenome and phenotype by analyzing gene expression.

In actuality, there have been many discussions regarding how an epigenome can affect a phenotype [4]. Because a gene can affect the phenotype through the regulation of gene expression, it is natural to expect that the epigenome can also affect the phenotype through the regulation of gene expression [5]. The epigenome is even expected to be heritable and thus affect phenotypes through generations [6]. In this field, the relation between the epigenome and phenotype has been comprehensively investigated; through regulation of gene expression, epigenetic mechanisms have the potential to determine and alter cell phenotypes, and epigenetic mechanisms also mediate dosage compensation, chromosomal silencing, and imprinting [7]. In this regard, castes of social insects are expected to be affected by various epigenomic alterations.

Some pioneering studies in this field have been conducted [8, 9], but statistical analyses in these studies have not been satisfactory, for example,

1. Gene ontology (GO) enrichment analyses had an insufficiently small false discovery rate (FDR): <0.5 [8];
2. identification of differentially expressed genes (DEGs) was performed at insufficiently large *q*>0.6, corresponding to FDR <0.4 [9];
3. highly methylated regions significantly different between phenotypes (castes) were not identified [8].

Even though these issues do not always reduce quality of the studies, addressing them should increase the confidence in the conclusions.

Inadequate statistical analyses may be due to disregarding the multivariate nature of variables. All the performed statistical analyses have been single–gene–based, meaning that group behaviors were considered only *after* identification of genes. Recently, I proposed a principal component analysis (PCA)-based unsupervised feature extraction (FE) as a method that can perform multivariate analysis *before* gene selection and applied it to various bioinformatic problems [10–31]. Therefore, applying PCA-based unsupervised FE to the analysis of datasets may yield more reliable results. PCA-based unsupervised FE was also extended to tensor decomposition (TD) to integrate multiway [32–34] and multiview [35–37] datasets. TD-based unsupervised FE applied to the integrated analysis of gene expression and methylation profiles may allow for identification of relations between gene expression and methylation, essential for identification of the mechanisms behind epigenetic regulation of phenotype development.

## Methods

**Fig. 1:**
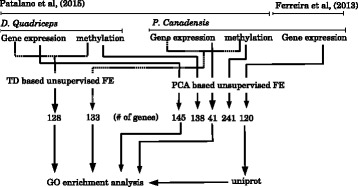
A flow chart showing the design of this study. Three gene expression profiles and two methylation profiles were retrieved from two studies on two social insect species, *Polistes canadensis* and *Dinoponera quadriceps*. All of them were processed by PCA-based unsupervised FE, whereas two pairs of gene expression and methylation profiles were analyzed by TD-based unsupervised FE. Differential expression between castes was analyzed in seven obtained gene sets. Analysis of GO term enrichment was performed on three sets of genes derived from gene expression analysis and on two sets of genes generated by TD-based unsupervised FE. The full list of the selected genes is presented in Additional file 1

A flow chart showing the experimental design is presented in Fig. 1.

### Gene expression and methylation profiles

All gene expression and methylation profiles [8] were retrieved from Gene Expression Omnibus (GEO). Gene expression profiles of *P. canadensis* and *D. quadriceps* are available as Supplementary Files in GEO ID GSE59525: GSE59525_RPKM_Pcan.txt.gz and SE59525_RPKM_Dqua.txt.gz. Methylation profiles of *P. canadensis* and *D. quadriceps* are in GSM14388XX_PcanYYY_CX.txt.gz and GSM14388XX_DquaYYY_CX.txt.gz, which are also available as Supplementary Files in GEO ID GSE59525 (XX and YYY are presented in Table 1). An additional gene expression profile of *P. canadensis* [9], 13059_2012_3057_MOESM9_ESM.CSV, was retrieved from the supplementary file presented in the study (Additional file 9). Gene expression values were used as-is, but methylation profile values were integrated so that they represented the relative methylation within genes. Assuming *m*_*s*1_ and *m*_*s*2_ are methylation and nonmethylation values respectively at locus *s*, then the relative methylation within the *i*th gene can be defined as

$$\frac{\sum_{s \in i} m_{s1}}{\sum_{s \in i} (m_{s1} +m_{s2})}, $$

where $\sum _{s \in i}$ is taken over *s* bases within DNA sequences corresponding to the *i*th gene body.

**Table 1:** The list of files containing methylation profiles retrieved from GEO

XX	YYY	XX	YYY	Description
*P. canadensis*		*D. quadriceps*		
50	_Synthetic	57	_Synthetic	Control
51	21Q	58	1AQ	Queen 1st replicate
52	43Q	59	2AQ	Queen 2nd replicate
53	75Q	60	3AQ	Queen 3rd replicate
54	26W	61	1CW	Worker 1st replicate
55	42W	62	3CW	Worker 2nd replicate
56	76W	63	3DW	Worker 3rd replicate

### PCA-based unsupervised FE

**Fig. 2:**
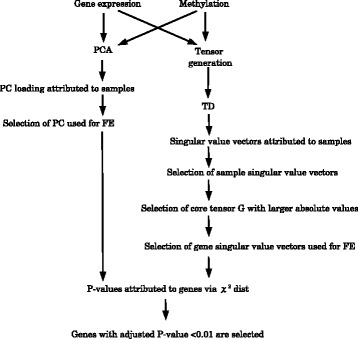
A flow chart of PCA- and TD-based unsupervised FE. Gene expression and methylation profiles were examined by PCA or TD. For PCA, gene expression and methylation profiles were processed separately, whereas TD was applied after generating a tensor from them. For PCA, principal component (PC) loading attributed to samples was studied and selected for FE. Because PC loadings and PC scores attributed to genes show one-to-one correspondence, PC scores corresponding to the selected PC loadings were subjected to FE. For TD, one-sample singular value vectors used for FE were selected. Afterwards, during analysis of core tensors, *G*, gene singular value vectors associated with *G*s with larger absolute values were selected. By means of the identified PC scores or gene singular value vectors, P-values were determined for genes, assuming a *χ*^2^ distribution, and genes associated with adjusted P-values less than 0.01 were finally selected

A flow chart showing PCA- and TD-based unsupervised FE is presented in Fig. 2.

#### PCA

Assume that *N*×*M* matrix *X* represents gene expression or methylation of the *i*th gene in the *j*th sample, $x_{ij} \in \mathbb {R}^{N \times M}$, which can be standardized as $\sum _{i} x_{ij} =0, \sum _{i} x^{2}_{ij} = N$. The *k*th PC score, $u_{ki} \in \mathbb {R}^{\min (N,M) \times N}$, attributed to the *i*th gene can be obtained as the *i*th component of the *k*th eigenvector *u*_*k*_ of *XX*^*T*^, where *X*^*T*^ is the transposed matrix of *X*, so that

$$XX^{T} {\boldsymbol{u}}_{k} = \lambda_{k} {\boldsymbol{u}}_{k}, $$

where *λ*_*k*_ is the *k*th eigenvalue. The *k*th PC loading, $v_{kj} \in \mathbb {R}^{{\min (N,M)} \times M}$, attributed to the *j*th sample can be obtained as the *j*th component of the *k*th vector *v*_*k*_, which is defined as

$${\boldsymbol{v}}_{k} = X^{T} {\boldsymbol{u}}_{k}. $$

This is also the *k*th eigenvector of matrix *X*^*T*^*X* because

$$X^{T} X {\boldsymbol{v}}_{k} = X^{T} X X^{T} {\boldsymbol{u}}_{k} = X^{T} \lambda_{k} {\boldsymbol{u}}_{k} = \lambda_{k} {\boldsymbol{v}}_{k}. $$

#### PCA-based unsupervised FE

To carry out FE by PCA, PC loadings, *v*_*k*_, of interest that can be used for FE must be identified, and there are several approaches. Suppose that *Ω* represents a set of *k*s of the identified *v*_*k*_s. Then, gene *i*, primarily contributing to the *k*th (*k*∈*Ω*) PC score, *u*_*k*_, should be identified, and this task can be accomplished by selecting the outliers within the space spanned by the PC scores:

$${span}({\boldsymbol{u}}_{k} : k \in \Omega).$$

*u*_*ki*_(*k*∈*Ω*,1≤*i*≤*N*) was assumed to follow a multiple normal distribution, and *P*-values, *P*_*i*_s, were attributed to each gene *i* via the *χ*^2^ distribution,

1 $$ P_{i} = P_{\chi^{2}} \left [>\sum_{k \in \Omega} \left (\frac{u_{ki}}{\sigma_{k}}\right)^{2} \right ],   $$

where $\phantom {\dot {i}\!}P_{\chi ^{2}}[>x]$ represents the cumulative probability of the *χ*^2^ distribution that the argument is greater than *x*, whereas *σ*_*k*_ is standard deviation. Afterwards, genes *i*s, associated with the Benjamini–Hochberg criterion–adjusted *P*_*i*_s [38] lower than the threshold value, e.g., 0.01, were selected as outliers.

### TD-based unsupervised FE

When two sets of experimental factors are affecting each sample, e.g., tissues and diseases, gene expression and methylation profiles should be presented as a tensor $x_{ij\ell } \in \mathbb {R}^{N \times M \times L}$, where *j* and *ℓ* correspond to the tissue and disease, respectively.

#### Equivalence of PCA and singular value decomposition (SVD)

PCA can be extended to a tensor as follows. PCA is known to be equivalent to SVD,

2 $$ U^{T} X V = \Lambda,   $$

where *U* and *V* are *N*×*M* and *M*×*M* orthogonal matrices, respectively. *Λ* represents a *M*×*M* diagonal matrix. Assume that *U*=(***u***_1_,…,***u***_*M*_) and *V*=(***v***_1_,…,***v***_*M*_). The diagonal component of *Λ* can be written as *λ*_*k*_. Then, using ***v***_*k*_=*X*^*T*^***u***_*k*_ and *XX*^*T*^***u***_*k*_=*λ****u***_*k*_,

$$U^{T} X V = U^{T} X X^{T} U = U^{T} \Lambda U = \Lambda. $$

Therefore, *U* and *V* composed of PC scores and loadings satisfy Eq. (2). Subsequently,

$$U U^{T} X V V^{T} = X = U \Lambda V^{T}, $$

or this relation can be written as

3 $$ x_{ij} = \sum_{k} \lambda_{k} u_{ki} v_{kj}.   $$

#### Extension to TD

Equation (3) can be easily generalized to TD [32] by extending the matrix to a tensor,

$$x_{ij\ell} = \sum_{k_{1}=1}^{N} \sum_{k_{2}=1}^{M} \sum_{k_{3}=1}^{L} G(k_{1},k_{2},k_{3})u_{k_{1}i}u_{k_{2}j}u_{k_{3} \ell}, $$

where $u_{k_{1}i} \in \mathbb {R}^{N \times N}$, $u_{k_{2}j} \in \mathbb {R}^{M \times M}$, $u_{k_{1}\ell } \in \mathbb {R}^{L \times L}$ and $U_{K_{i}}= (\text {\boldmath {u}}_{1},\ldots,\text {\boldmath {u}}_{K_{i}})$ with (*K*_1_,*K*_2_,*K*_3_)=(*N*,*M*,*L*) were assumed to be orthogonal matrices. Hereafter, $U_{K_{i}}$ and $\text {\boldmath {u}}_{k_{i}}$ are referred to as singular value matrix and singular value vector, respectively. Given that the core tensor, $G(k_{1},k_{2},k_{3}) \in \mathbb {R}^{N \times M \times L}$, is as large as *x*_*ijk*_, this situation represents an overcomplete problem, i.e., there is no unique decomposition. In this study, higher-order SVD (HOSVD) [39], which is known to frequently give a global minimum [40], was employed to perform TD, assuming $\sum _{i} x_{ij\ell } =0, \sum _{i} x^{2}_{ij\ell } = N$, as in the PCA cases.

#### TD-based unsupervised FE

First, (*k*_2_,*k*_3_) of interest, which were attributed to samples, were selected, and, as for PCA, there are different approaches. Next, *G*(*k*_1_,*k*_2_,*k*_3_)s associated with selected *k*_2_,*k*_3_ were ranked based on their absolute values, and by means of top-ranked *k*_1_s with the set defined as *Ω*, the same procedure applied to Eq. (1) was repeated by replacing PC score *u*_*ki*_ with a gene singular value vector, $u_{k_{1}i}$. Currently, we do not have any specific criterion specifying how many *k*_1_s should be considered.

#### TD-based unsupervised FE for integrated analysis of multi-omics data

For two distinct omics datasets, such as a gene expression profile, *x*_*ij*_, and a methylation profile, *x*_*iℓ*_, TD-based unsupervised FE can be used for the integrated analysis by generating a tensor,

$$x_{ij\ell} = x_{ij}x_{i\ell}, $$

to which TD-based unsupervised FE can be applied [35]. The subsequent procedure was the same as the standard one described in the previous subsection.

### GO enrichment analysis

To perform GO enrichment analysis of the previously described dataset [8], the list of genes associated with GO terms was downloaded [41]: PCAN.v01.GO.tsv for *P. canadensis* and DQUA.v01.GO.tsv for *D. quadriceps*.

Because genes presented in the second study we used [9] were not fully annotated but contained protein sequence gene IDs, a list of gene IDs was uploaded to UniProt [42] and GO term associations were downloaded. GO enrichment analyses were performed on the retrieved list.

Fisher’s exact test was selected for evaluation of the overlaps between the set of provided genes and genes associated with a specific GO term. The obtained *P*-values were adjusted in accordance with the Benjamini–Hochberg criterion [38]. GO terms associated with adjusted *P*-values less than 0.01 were selected.

## Results

### PCA-based unsupervised FE applied to the dataset provided by Patalano et al. [8]

PCA-based unsupervised FE was performed on the dataset presented in another study [8].

#### The methylation profile

**Fig. 3:**
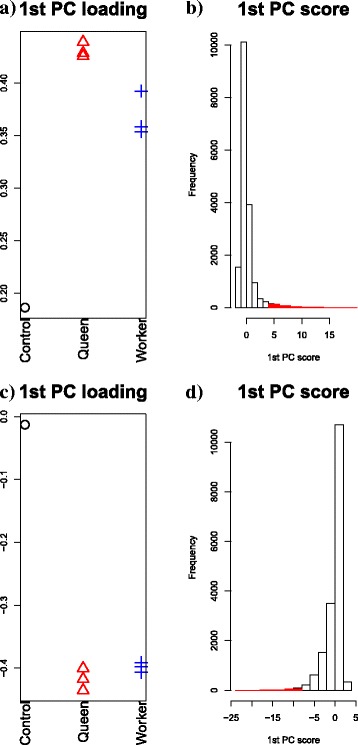
The results of PCA performed on methylation profiles. PCA-based unsupervised FE was tested on methylation profiles. **a** and **b**: *P. canadensis*. **c** and **d**: *D. quadriceps*. **a** and **c**: A boxplot of PC loadings *v*_1_, (**b**) and (**d**): A PC score histogram, *u*_1_. Red areas in (**b**) and (**d**) represent selected genes

PCA-based unsupervised FE was applied to the methylation profiles of *P. canadensis* and *D. quadriceps*. In Fig. 3(a) and (c), the first PC loadings, *v*_1_, are presented, attributed to seven samples, comprising one control, three queen samples, and three worker samples of *P. canadensis* and *D. quadriceps* each. For both, *v*_1_ mainly denotes the difference between the control and queen or worker samples. In Fig. 3(b) and (d), the histogram of the first PC score, *u*_1_, attributed to genes is presented, and red areas represent the selected genes, with adjusted *P*-values computed using Eq. (1) lower than 0.01 (241 and 138 selected genes for *P. canadensis* and *D. quadriceps*, respectively). Because in Fig. 3(a) hypermethylated genes are presented, and the results in Fig. 3(b) suggest that only the genes with positive PC scores are selected, all the selected genes were found to be hypermethylated. Similarly, because in Fig. 3(c), hypomethylation is presented, and the results in Fig. 3(d) indicate that only the genes with negative PC scores are selected, all the selected genes were found to be associated with hypermethylation as well. This finding is in agreement with the results of the other study [8].

Patalano et al. [8] did not find genes associated with the emergence of distinct methylation patterns between queens and workers, but the results presented in Fig. 3(a) and (c) show minor differences between queens and workers, suggesting that the selected genes may have different methylation profiles between queens and workers overall. Three statistical tests were carried out to analyze the differences between queens and workers (Table 2) and demonstrated that PCA-based unsupervised FE could identify gene-associated methylation patterns between queens and workers, unlike the analyses in the other study [8]. Given that in Fig. 3(a) and (c), the upregulation and downregulation of methylation in queens are presented, whereas in Fig. 3(b) and (d), genes associated with positive and negative PC scores are shown, the selected genes were found to be associated with hypermethylation in queens. This finding suggests that hypermethylated genes are associated with relative hypermethylation in queens.

**Table 2:** Statistical tests for differences in the methylation rates of selected genes between queens and workers

	*t*	Wilcox	KS
*P. canadensis*	8.59×10^−5^	3.10×10^−3^	1.83×10^−4^
*D. quadriceps*	1.11×10^−2^	5.88×10^−3^	1.75×10^−3^

*t*: the *t* test, Wilcox: the Wilcoxon rank sum test, KS: the Kolmogorov–Sinai test, all two-sided

#### Gene expression

**Fig. 4:**
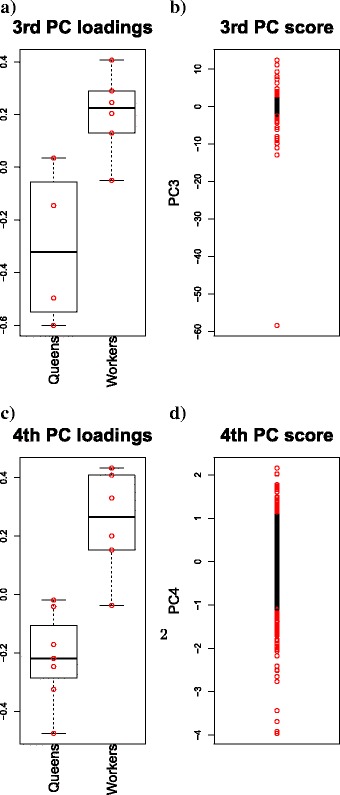
Results of gene expression PCA. PCA-based unsupervised FE was applied to the gene expression profiles. **a** and **b**: *P. canadensis*. **c** and **d**: *D. quadriceps*. **a** and **c**: A boxplot of PC loadings *v*_1_, (**b**) and (**d**): A distribution of PC scores *u*_1_. Red areas in (**b**) and (**d**) represent the selected genes

PCA-based unsupervised FE was performed on the gene expression profiles of *P. canadensis* and *D. quadriceps*. Because the gene expression profile of *P. canadensis* was log2-ratio converted, it was scaled back to the original one as $\phantom {\dot {i}\!}2^{x_{ij}}$ before the application of PCA. In Fig. 4(a) and (c), *v*_3_ and *v*_4_ are presented, showing the most significant differences in gene expression levels between queens and workers of *P. canadensis* and *D. quadriceps*, respectively. In Fig. 4(b) and (d), the distributions of *v*_3_ and *v*_4_ for *P. canadensis* and *D. quadriceps*, respectively, are depicted. Red areas represent the selected genes, with adjusted *P*-values, determined using Eq. (1), lower than 0.01 (41 and 145 genes for *P. canadensis* and *D. quadriceps*, respectively).

To determine whether the expression of selected genes differs between queens and workers, three statistical tests were carried out (Table 3). The majority (five of six) of the applied tests confirmed that expression of the selected genes differs between queens and workers of *P. canadensis* and *D. quadriceps*, in line with the findings reported elsewhere [8].

**Table 3:** Statistical tests for differences in expression of the selected genes between queens and workers

	*t*	Wilcox	KS
*P. canadensis*	4.37×10^−4^	0.07	6.45×10^−3^
*D. quadriceps*	1.73×10^−12^	2.24×10^−12^	5.26×10^−12^

*t*: the *t* test, Wilcox: the Wilcoxon rank sum test, KS: the Kolmogorov–Sinai test; all two-sided

### PCA-based unsupervised FE applied to the dataset provided by Ferreira et al. [9]

**Fig. 5:**
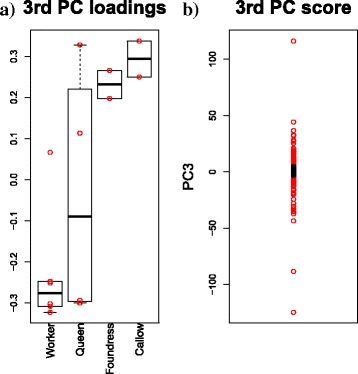
The results of PCA applied to gene expression profiles. PCA-based unsupervised FE was performed on the gene expression profiles obtained from the study by Ferreira et al. **a**: A boxplot of PC loadings *v*_1_. **b**: A distribution of PC scores *u*_1_. Red areas in panel (**b**) represent 120 selected genes. PC3 > 0: 77 genes, PC3 < 0: 43 genes

PCA-based unsupervised FE was performed on the expression profiles reported in another study [9]. In Fig. 5(a), the third PC loadings, *v*_3_, are presented, which demonstrate the most significant class dependence based on categorical regression (ANOVA). In Fig. 5(b), the distribution of the third PC score, *u*_3_, is shown, where the red areas represent the selected genes (120 genes associated with the adjusted *P*-values, as determined by means of Eq. (1), lower than 0.01). Because this situation corresponds to *q*>0.99 in the analysis provided by Ferreira et al., we demonstrated that we were able to identify a set of genes more significant than those identified in the other study, where *q* was > 0.6.

To test whether the selected genes reflect the class-specific up- and downregulation of expression, three statistical tests were conducted (Table 4). We separated genes into two classes based on the sign of *u*_3*i*_, to identify the upregulation or downregulation of these genes for further comparisons. As shown in Table 4, 120 selected genes were found to be significantly upregulated or downregulated, excluding the upregulation of genes in Foudress, with the smallest number of genes identified in the other study. Therefore, we successfully identified genes that manifest class-specific upregulation or downregulation. Furthermore, the genes identified here are common for all four classes, while those identified in the other study differed between the comparisons.

**Table 4:** Statistical tests for upregulation and downregulation of gene expression in four categories vs. others

	PC3		*t*	Wilcox	KS
Worker vs others	+	down	3.96×10^−5^	∗	∗
	−	up	0.22	∗	∗
Queen vs others	+	up	0.36	0.10	8.87×10^−4^
	−	down	0.32	0.69	4.69×10^−3^
Foundress vs others	+	up	0.05	0.13	0.17
	−	down	0.11	3.49×10^−10^	6.27×10^−11^
Callow vs others	+	up	0.99	∗	∗
	−	down	1.14×10^−6^	2.77×10^−7^	3.34×10^−6^

∗: < 2.2×10^−16^. *t*: the *t* test, Wilcox: the Wilcoxon rank sum test, KS: the Kolmogorov–Sinai test, all are two-sided

### TD-based unsupervised FE applied to the integrated analysis of gene expression and methylation profiles

By PCA-based unsupervised FE, we successfully identified genes associated with gene expression and methylation profiles showing significant differences between castes. Genes found by means of gene expression profiles and methylation profiles did not overlap. Fisher’s test results yielded an odds ratio lower than 1.0 (data not shown because of negative results). This finding indicated that the genes identified using different datasets are quite distinct. Therefore, it is difficult to understand the mechanisms by which the epigenetic modifications affect gene expression and regulate phenotype development.

One may wonder if a gene expression alteration must not always be associated with altered methylation. Nevertheless, many authors have employed the strategy where genes associated with both altered gene expression and methylation are sought, to identify biologically important genes. Heng et al. [43, 44] have tried to find genes associated with both altered gene expression and methylation to discover genes crucial for breast cancers. Li et al. [45] have attempted to find genes associated with both altered gene expression and methylation to identify key genes in severe oligozoospermia. Mallik et al. [46] have looked for genes associated with both altered gene expression and methylation for tumor prediction. These are only a few examples of studies that involve the association of altered gene expression and methylation. Thus, altered gene expression and promoter methylation may have to be considered together to identify genes specific for castes of social insects, too.

**Fig. 6:**
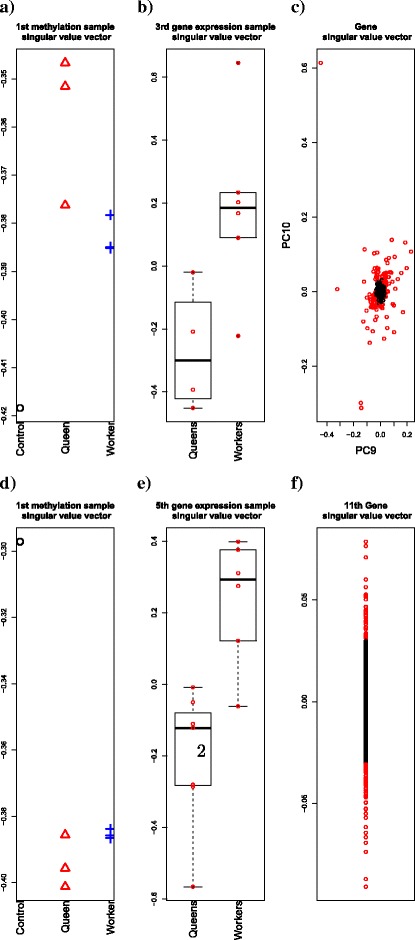
TD analysis of gene expression profiles. TD-based unsupervised FE was applied to gene expression and methylation profiles of *P. canadensis* (**a**, **b**, **c)**and *D. quadriceps* (**d**, **e**, **f**). **a**: The first sample singular value vector for methylation profiles $u_{k_{3}\ell }$ (with *k*_3_=1), (**b**) A boxplot of the third sample singular value vectors for gene expression profiles, $u_{k_{2}j}$ (with *k*_2_=3). **c** The ninth and 10th gene singular value vector, $u_{k_{1}i}$ (with 9≤*k*_1_≤10). Red areas in (**c**) represent 133 selected genes. **d**: The first sample singular value vector for methylation profiles, $u_{k_{3}\ell }$ (at *k*_3_=1). **e** A boxplot of the fifth sample singular value vectors for gene expression profiles, $u_{k_{2}j}$ (at *k*_2_=5). **f** The 11th gene singular value vector, $u_{k_{1}i}$ (at *k*_1_=11). Red areas in (**f**) represent 128 selected genes

To explore the possibility of correlating gene expression and methylation profiles via our strategy, we applied TD-based unsupervised FE to tensor *x*_*ijℓ*_=*x*_*ij*_*x*_*iℓ*_, where *i* represents a gene, *x*_*ij*_ is the gene expression of the *j*th sample, and *x*_*iℓ*_ denotes methylation of the *ℓ*th sample. In Fig. 6(a), the first sample singular value vector for gene expression, $u_{k_{2}j}$ (at *k*_2_=1) for *P. canadensis* is shown, which has the most significant dependence upon class labels based upon categorical regression. Because the presented results are similar to those shown in Fig. 3(a), TD-based unsupervised FE was demonstrated to successfully generate biologically relevant singular value vectors for gene expression, $u_{k_{2}j}$ (at *k*_2_=1). Similarly, results depicted in Fig. 6(b) represent the third sample singular value vectors for the methylation profiles of *P. canadensis*, $u_{k_{3}\ell }$ (with *k*_3_=3), which were also found to be similar to those presented in Fig. 4(a). This finding indicates that TD-based unsupervised FE can successfully generate biologically relevant sample singular value vectors.

Next, we aimed to identify core tensor *G*(*k*_1_,*k*_2_,*k*_3_) (at (*k*_2_,*k*_3_)=(1,3)) associated with the larger absolute values, to select *k*_1_s used for the gene selection based on *P. canadensis* profiles (Table 5). Given that *G*(*k*_1_,*k*_2_,*k*_3_) (with 9≤*k*_1_≤10,(*k*_2_,*k*_3_)=(1,3)) were shown to be top-ranked, the ninth and 10th singular value vectors, $u_{k_{1}i}$ (with 9≤*k*_1_≤10) were used for the selection of 133 genes, associated with the adjusted *P*-values, determined via Eq. (1), lower than 0.01 (Fig. 6(c)).

**Table 5:** The top 10 core tensors, *G*, with large absolute values

*P. canadensis*		*D. quadriceps*	
*k*_1_	*G*(*k*_1_,*k*_2_,*k*_3_)	*k*_1_	*G*(*k*_1_,*k*_2_,*k*_3_)
	(*k*_2_,*k*_3_)=(1,3)		(*k*_2_,*k*_3_)=(1,5)
9	−79.8	11	−54.8
10	75.4	12	4.1
7	−61.4	25	3.4
11	38.4	2	−2.9
5	−23.4	23	2.8
4	−16.0	9	2.4
12	−11.9	20	−2.2
1	−5.4	8	2.2
13	5.4	10	−1.7
6	−4.5	22	−1.4

To determine whether the selected genes show differential expression and methylation between workers and queens, three statistical tests were applied (Table 6). We observed that 133 selected genes have significant differences in expression and methylation between the samples under study. Therefore, using TD-based unsupervised FE, we successfully identified a set of genes that have both differential gene expression and methylation between queens and workers; this accomplishment was not possible with the PCA-based unsupervised FE performed individually on gene expression and methylation profiles.

**Table 6:** Statistical tests of the differences (between queens and workers) in gene expression and methylation

		*t*	Wilcox	KS
*P. canadensis*	gene expression	1.71×10^−3^	1.89×10^−2^	0.08
	methylation	1.74×10^−4^	5.06×10^−3^	1.02×10^−3^
*D. quadriceps*	gene expression	2.73×10^−12^	9.05×10^−12^	4.41×10^−11^
	methylation	0.3757	0.7163	0.4413

The genes identified by TD-based unsupervised FE were analyzed by *t* (the *t* test), Wilcox (the Wilcoxon rank sum test), and KS (the Kolmogorov–Sinai test), all two-sided

In Fig. 6(d), the first sample singular value vector for gene expression, $u_{k_{2}j}$ (with *k*_2_=1) for *D. quadriceps* is presented, which was shown to have the most significant dependence upon class labels based on categorical regression. Because these results were shown to be similar to those presented in Fig. 3(c), TD-based unsupervised FE was demonstrated to successfully generate biologically relevant sample singular value vectors of gene expression, $u_{k_{2}j}$ (with *k*_2_=1). Similarly, in Fig. 6(e), the fifth sample singular value vectors for the methylation profile of *D. quadriceps* are depicted, $u_{k_{3}\ell }$ (with *k*_3_=5), which were found to be similar to those in Fig. 4(c). This finding indicates that TD-based unsupervised FE can lead to successful generation of biologically relevant sample singular value vectors.

Furthermore, we aimed to identify the core tensor *G*(*k*_1_,*k*_2_,*k*_3_) (at (*k*_2_,*k*_3_)=(1,5)) associated with the increased absolute values, to identify the *k*_1_s used for the gene selection in *D. quadriceps* datasets (Table 5). Because *G*(*k*_1_,*k*_2_,*k*_3_) (with (*k*_1_,*k*_2_,*k*_3_)=(11,1,5)) is top-ranked, the 11th gene singular value vectors, $u_{k_{1}=11,i}$, were employed for the selection of 128 genes associated with the adjusted *P*-values, determined using Eq. (1), lower than 0.01 (Fig. 6(f)).

To confirm that the selected genes are associated with differential gene expression and methylation between workers and queens, three statistical tests were applied (Table 6). We demonstrated that the 128 selected genes are associated with significant differences in gene expression but not methylation between queens and workers. Therefore, by TD-based unsupervised FE, we successfully found a set of genes associated with the differential gene expression but not methylation between queens and workers, suggesting that this analysis was not successful in the case of *D. quadriceps*.

### GO enrichment analysis

We demonstrated that PCA- and TD-based unsupervised FE can be used for the successful identification of genes associated with differential gene expression and methylation between workers and queens, but these results may be improved by showing that these sets of genes are biologically relevant as well. GO enrichment analysis was performed on three sets of genes selected on the basis of the gene expression profiles obtained from other studies (two *P. canadensis* datasets and one *D. quadriceps* dataset [8, 9], Table 7).

**Table 7:** GO enrichment analysis of the genes selected using gene expression profiles

* P. canadensis*	
Dataset provided by Patalano et al. [8]	
Results obtained in this study (PCA)	
GO:0005319	Lipid transporter activity
GO:0006869	Lipid transport
Results obtained in this study (TD)	
GO:0005319	Lipid transporter activity
GO:0005811	Lipid particle
GO:0006869	Lipid transport
Patalano et al. [8]	
No enrichments	
Dataset provided by Ferreira et al. [9]	
Results obtained in this study (PCA)	
GO:0004129	Cytochrome-c oxidase activity
GO:0003735	Structural constituent of ribosome
GO:0006412	Translation
GO:0005743	Mitochondrial inner membrane
GO:0008137	NADH dehydrogenase (ubiquinone) activity
*D. quadriceps*	
Dataset provided by Patalano et al. [8]	
Results obtained in this study (PCA)	
GO:0005506	Iron ion binding
GO:0009055	Electron carrier activity
GO:0016705	Oxidoreductase activity, acting on paired donors,
	with incorporation or reduction of molecular oxygen
GO:0020037	heme binding
GO:0055114	Oxidation-reduction process
Results obtained in this study (TD)	
No enrichments	
Patalano et al. [8]	
GO:0003735	Structural constituent of ribosome
GO:0005622	Intracellular
GO:0005840	Ribosome
GO:0005842	Cytosolic large ribosomal subunit
GO:0006412	Translation

In contrast to the results of the other study on *P. canadensis* gene expression profiles [8], which showed no enrichment data, by means of the same dataset, we identified two enriched GO terms using the results obtained by PCA-based unsupervised FE. In the TD analysis, the number of enriched GO terms increased to three. These results indicate that we successfully performed the integrated analysis via TD-based unsupervised FE on *P. canadensis* datasets.

For *D. quadriceps* profiles, both genes identified by Patalano et al. [8] and those selected by PCA-based unsupervised FE were found to be associated with the same number of enriched GO terms, five, although the identified terms were not identical. Nevertheless, in the TD analysis, we did not observe any enrichment, and this result coincides with the fact that TD-based unsupervised FE failed to identify genes associated with differences in methylation profiles between queens and workers (Table 6).

Finally, in the analysis of genes identified by PCA-based unsupervised FE in the dataset provided by Ferreira et al. [9], five enriched GO terms were identified as well. Therefore, PCA- and TD-based unsupervised FE methods were shown to successfully identify biologically relevant sets of genes associated with significant enrichment in GO terms.

## Discussion

### Biological importance of the obtained results

We observed some instances of enrichment of GO terms, but the biological importance of our results should be examined further. In *P. canadensis* analysis, GO terms related to lipid transport were found to be enriched. Recently, Ament et al. [47] reported that worker honey bees undergo a socially regulated, highly stable lipid loss as part of their behavioral maturation. Given that *P. canadensis* is a bee species as well, the observed GO term enrichment of genes with differential gene expression and methylation profiles may be promising. Altered methylation of these genes may induce changes in gene expression that result in a highly stable lipid loss. This arrangement may enable the coexistence of multiple phenotypes.

In contrast, oxidation-reduction processes, regulated by genes expressed differently between queens and workers of *D. quadriceps*, have been reported to be upregulated in the queens of multiple ant species [48]. Because *D. quadriceps* is an ant species, this means that we correctly identified DEGs between workers and queens, via the proposed strategy. In addition to the heme-binding–associated genes, shown to be differently expressed between queens and workers of *D. quadriceps*, they were associated with aberrant methylation in termites [49], another species of social insects.

Furthermore, oxidoreductase activity–related genes, found to be differently expressed between queens and workers of *D. quadriceps*, have been reported to be expressed in the queens of honey bees too [50].

Alaux et al. [51] determined genes associated with electron carrier activity—shown to be differentially expressed between the queens and workers of *D. quadriceps* in this study as well—in another study, which analyzed the relation between aggressiveness and behavioral evolution in honey bees.

When genes of *D. quadriceps*, identified by Patalano et al. were analyzed, more general GO terms were obtained, e.g., those related to translation and ribosomes, which are unlikely to be related to social-insect–specific features. In contrast, identification of more specific GO terms and the results of other studies point to biological importance of the analyses presented here.

### Future directions

This paper shows the importance of integrated analyses of gene expression and promoter methylation for finding genes that might link castes of social insects to the epigenome. At the moment, only two species were investigated, but because castes of social insects have been examined in multiple species that do not belong to even the same family from the genetic point of view [52], this approach should be extended other species. Inclusion of more species may clarify how castes of social insects have evolved and have been maintained from the standpoint of epigenetics.

## Conclusions

Here, we tested newly developed PCA- and TD-based unsupervised FE for the analysis of gene expression and methylation profiles of *P. canadensis* and *D. quadriceps*. The issues observed in other studies were solved as follows:

1. GO enrichment analysis was performed successfully on *P. canadensis* gene expression profiles [8] with a strict criterion of FDR less than 0.01 (Table 7);
2. Genes found to be differentially expressed among four castes [9] were analyzed by means of a strict criterion of FDR less than 0.01 (Table 6);
3. Genes associated with differential methylation between queens and workers of *P. canadensis* were analyzed successfully [8] (Table 2).

Therefore, PCA- and TD-based unsupervised FE methods were successfully performed on ’omics datasets comprising gene expression and methylation profiles. The obtained sets of genes will help us understand how development of caste phenotypes is regulated epigenetically.

## Additional file

Additional file 1 Genes selected by PCA- and TD-based unsupervised FE. (XLSX 16 kb)

## Abbreviations

FE: Feature extraction
PC: Principal component
PCA: Principal component analysis
TD: Tensor decomposition
